# The complete mitochondrial genome of the swimming crab *Charybdis natator* (Herbst) (Decapoda: Brachyura: Portunidae) and its phylogeny

**DOI:** 10.1080/23802359.2017.1365639

**Published:** 2017-08-17

**Authors:** Xiaolong Yang, Hongyu Ma, Khor Waiho, Hanafiah Fazhan, Shuqi Wang, Qingyang Wu, Xi Shi, Cuihong You, Jianxue Lu

**Affiliations:** a Guangdong Provincial Key Laboratory of Marine Biotechnology, Shantou University, Shantou, China;; b East China Sea Fisheries Research Institute, Chinese Academy of Fishery Sciences, Shanghai, China

**Keywords:** *Charybdis natator*, gene arrangement, genome structure, mitochondrial genome, phylogeny

## Abstract

The complete mitochondrial genome of *Charybdis natator* (family Portunidae) was obtained using Illumina and Sanger dideoxy sequencing. This genome was a typically circular molecule with a length of 15,664 bp and encoded 13 protein-coding genes, 22 transfer RNA genes and 2 ribosomal RNA genes. The overall base composition of this mitogenome was 34.00% for A, 11.06% for G, 36.36% for T, and 18.58% for C, respectively, with a higher A + T content (70.36%). The gene composition and arrangement were accordant to the closely published species. The phylogenetic analysis suggested that *C. natator* had the closest relationship with *C. japonica*.

The marine crab, *Charybdis natator*, also known commonly as the ridged swimming crab, is a species of the family Portunidae that has a geographical distribution around the Indo-West Pacific, including China, Australia, Indonesia, Thailand, Malaysia, Singapore, and Japan (Wee and Ng 1995; Islam et al. 2000). Although *C. natator* is not as famous as other economically important portunids such as the mud crab genus *Scylla* (Ikhwanuddin et al. 2011; Waiho et al. 2016; Fazhan et al. 2017) and the blue swimming crab *Portunus pelagicus* (Azra and Ikhwanuddin 2015), the high meat yield makes it a possible candidate for future crab fisheries. The availability of molecular data, especially mitogenome, could be useful for understanding the population genetics and relationship of a species, and its evolution history (Behera et al. 2016). In the present study, the first complete mitochondrial genome DNA sequence of the swimming crab *C. natator* is determined and described.

Adult specimens were collected from a commercial crab market in Xiamen City, China (24.544821°N, 118.117269°E). Total genomic DNA was isolated from the muscle tissue and prepared in paired-end library which was then sequenced on an Illumina HiSeq 2000 platform. The mitogenome was assembled and compared with the complete mitogenome DNA of a closely related species (*Charybdis japonica*) (Liu and Cui 2010) using SeqMan software. The gaps in the mitochondrial genome of *C. natator* were filled by PCR and Sanger dideoxy sequencing with the designed primers based on the flanking sequences of the gaps.

The complete mitogenome sequence of *C. natator* was 15,664 bp in length (GenBank accession No. MF285241) and contained 13 protein-coding genes, 22 transfer RNA genes, 2 ribosomal RNA genes and 1 putative control region. Among the 37 genes, 23 genes were encoded by heavy strand, while the other genes were encoded by light strand. The gene arrangement and genome structure were accordant to those of the published species of Portunids. The overall base composition of the mitogenome was 34.00% for A, 11.06% for G, 36.36% for T, and 18.58% for C, respectively, with a higher A + T content (70.36%), just as in *Charybdis feriata* (Ma et al. 2015) and *C. japonica* (Liu and Cui 2010). The 13 protein-coding genes had a varying length from 162 to 1728 bp and encoded 3714 amino acids in total. The two rRNA genes were 1329 bp (*16S*) and 833 bp (*12S*) in length, respectively. The putative control region was 766 bp in length and located between 12S rRNA and *tRNA^Ile^*.

The phylogenetic position of *C. natator* was determined by comparison with the concatenated sequences of 12 mitochondrial protein-coding genes (not including *ND6*) of Decapoda species with published complete mitogenome sequences in GenBank database. As shown in Figure 1, *C. natator* clustered with its close congener *C. japonica* and *C. feriata*, as well as *Thalamita crenata*, and had the closest relationship with *C. japonica*. Meanwhile, there may be a genetically closer relationship between the genus *Charybdis* and *Thalamita*, which will be of great importance in identifying and understanding the evolution and phylogeny of *C. natator* and other decapod species.

**Figure 1.**
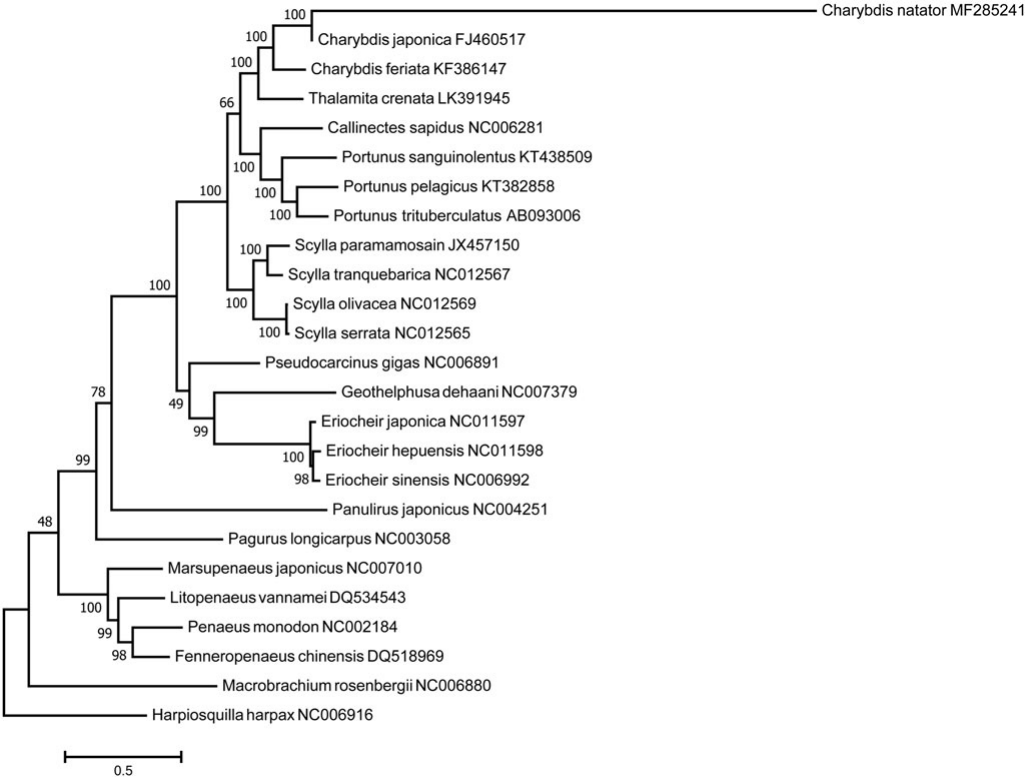
Phylogenetic tree of decapod relationships derived from maximum likelihood (ML) based on the linked sequences of 12 protein-coding gene with *Harpiosquilla harpax* (GenBank: NC006916) as an out-group. The number in each branch indicated the bootstrap value of ML analysis. GenBank accession numbers were indicated next to species designations.
